# Racism and Cancer Screening among Low-Income, African American Women: A Multilevel, Longitudinal Analysis of 2-1-1 Texas Callers

**DOI:** 10.3390/ijerph182111267

**Published:** 2021-10-27

**Authors:** Lynn N. Ibekwe, Maria Eugenia Fernández-Esquer, Sandi L. Pruitt, Nalini Ranjit, Maria E. Fernández

**Affiliations:** 1 Center for Health Promotion and Prevention Research, School of Public Health, The University of Texas Health Science Center at Houston, Houston, TX 77030, USA; Maria.E.Fernandez-Esquer@uth.tmc.edu (M.E.F.-E.); Maria.E.Fernandez@uth.tmc.edu (M.E.F.); 2 Department of Social and Behavioral Sciences, Harvard T.H. Chan School of Public Health, Harvard University, Boston, MA 02115, USA; 3 Kraft Center for Community Health, Massachusetts General Hospital, Boston, MA 02114, USA; 4 Department of Population and Data Sciences, The University of Texas Southwestern Medical Center, Dallas, TX 75390, USA; Sandi.Pruitt@UTSouthwestern.edu; 5 Harold C. Simmons Comprehensive Cancer Center, The University of Texas Southwestern Medical Center, Dallas, TX 75390, USA; 6 Michael and Susan Dell Center for Healthy Living, School of Public Health, The University of Texas Health Science Center at Houston–Austin Regional Campus, Austin, TX 78701, USA; Nalini.Ranjit@uth.tmc.edu

**Keywords:** cancer screening, racial discrimination, racial residential segregation, racism, African Americans, critical race theory, breast cancer, cervical cancer, colorectal cancer, 2-1-1

## Abstract

Although racism is increasingly being studied as an important contributor to racial health disparities, its relation to cancer-related outcomes among African Americans remains unclear. The purpose of this study was to help clarify the relation between two indicators of racism—perceived racial discrimination and racial residential segregation—and cancer screening. We conducted a multilevel, longitudinal study among a medically underserved population of African Americans in Texas. We assessed discrimination using the Experiences of Discrimination Scale and segregation using the Location Quotient for Racial Residential Segregation. The outcome examined was “any cancer screening completion” (Pap test, mammography, and/or colorectal cancer screening) at follow-up (3–10 months post-baseline). We tested hypothesized relations using multilevel logistic regression. We also conducted interaction and stratified analyses to explore whether discrimination modified the relation between segregation and screening completion. We found a significant positive relation between discrimination and screening and a non-significant negative relation between segregation and screening. Preliminary evidence suggests that discrimination modifies the relation between segregation and screening. Racism has a nuanced association with cancer screening among African Americans. Perceived racial discrimination and racial residential segregation should be considered jointly, rather than independently, to better understand their influence on cancer screening behavior.

## 1. Introduction

Cancer screening is a critical cancer prevention and control behavior that can help reduce cancer morbidity and mortality through prevention and early detection. Although screening has contributed to significant declines in cancer mortality in the last few decades [1], not all populations have benefited equally. In the United States, racial and ethnic minorities often suffer disproportionately from cancer as compared to their White counterparts [2,3,4]. In particular, African Americans, whose incidence of cancer is nearly the same as the incidence among Whites, are 14% more likely to die from cancer compared to their White counterparts [5]. Such disparities are attributed, in part, to racial differences in the stage of diagnosis between African Americans and Whites, which is often influenced by lower cancer screening and follow-up rates among African Americans [6,7]. Notably, Texas has among the lowest screening rates for cervical, breast, and colorectal cancer in the country, with cervical and colorectal cancer screening rates among African Americans at 69.3% and 63.7%, respectively, compared to 78.8% and 68.6%, respectively, among Whites [8]. While rates of breast cancer screening are comparable across African Americans and Whites (74.4% vs. 74.5%), the statewide prevalence estimates do not do justice to spatial differences in the extent of disparities [9,10,11,12,13,14].

Research on disparities in health, including health behavior, suggests that racism is an important determinant of inequities faced by African Americans. In particular, indicators of racism, such as perceived racial discrimination [15,16,17,18,19,20,21] and racial residential segregation [22,23,24,25], are associated with adverse health outcomes, including cancer incidence and mortality and lack of participation in healthful behaviors. The literature on the relation between these indicators of racism and cancer prevention and control behaviors, such as cancer screening, is limited, especially as it relates to segregation and cancer screening. Among the literature that does exist, findings are mixed. For example, perceived racial discrimination was reported as both negatively [26,27,28,29,30] and positively [31] associated with cancer screening, while some studies found no significant association [27,28,31,32,33,34,35]. These mixed findings are likely due to methodological limitations, such as inconsistent measurement of perceived racial discrimination [36,37,38,39,40], lack of longitudinal analyses [27,33], and limited investigation of both individual-level and neighborhood-level mechanisms that underlie these relations [22,41,42]. There are similar mixed findings in the few studies that have examined the association between racial residential segregation and cancer screening. One study reported a negative association between living in a segregated neighborhood and mammography use [43], another study reported a negative association with colorectal cancer screening [44], and the third study reported a positive association with mammography use in some states, while a negative association existed in other states [45]. Such differences across studies may be the result of differences in study population characteristics or experiences that potentially modify the relation between segregation and cancer screening among African Americans. For example, there is a reported association between segregation and perceived racial discrimination [46,47]. Longitudinal evidence suggests that African Americans who live in neighborhoods with a lower percentage of African Americans are significantly more likely to report racial discrimination compared to those who live in neighborhoods with a higher percentage of African Americans [46,47]. Despite this association, and that segregation and perceived racial discrimination are significant determinants of health, few studies examine the potential interactive impact of segregation and perceived racial discrimination on health behavior among African Americans [22,41,42]. Such evidence may help to explain the mixed results seen when examining independent associations between each of these indicators of racism and cancer screening among African Americans.

The purpose of this study was to, first, examine independent associations between perceived racial discrimination and racial residential segregation with any cancer screening completion (Pap test, colorectal cancer screening, and/or mammography) among a sample of low-income, medically underserved African American women, a group at high-risk for cancer-related disparities. We, then, explored the extent to which perceived racial discrimination modifies the relation between racial residential segregation and any cancer screening completion. Because this study uses longitudinal and multilevel data, it has several advantages over the other studies described above. Based on our conceptual framework, derived from the extant literature [48,49,50,51], we expected a negative relation between any cancer screening completion and both indicators of racism—racial discrimination (Hypothesis 1) and residential segregation (Hypothesis 2). As a hypothesis-generating aim, we also explored the extent to which racial discrimination modified the relation between residential segregation and any cancer screening completion. 

## 2. Materials and Methods

### 2.1. Study Design

We conducted a multilevel longitudinal study that examined the independent and interactive effect of baseline levels of perceived racial discrimination and racial residential segregation on the uptake of cancer screening at follow-up (3–10 months after completing the baseline survey) among a sample of African American women. Data for the current study were collected as part of an intervention trial, the 2-1-1 Cancer Prevention and Control Phone Navigation Study (hereafter referred to as “parent study”), and were merged with neighborhood-level (i.e., census tract) data from the US Census Bureau. The parent study (Study HSC-SPH-10-0241) and current study (Study HSC-SPH-20-1103) were approved by the Committee for the Protection of Human Subjects at The University of Texas Health Science Center at Houston.

### 2.2. Conceptual Framework

**Figure 1:**
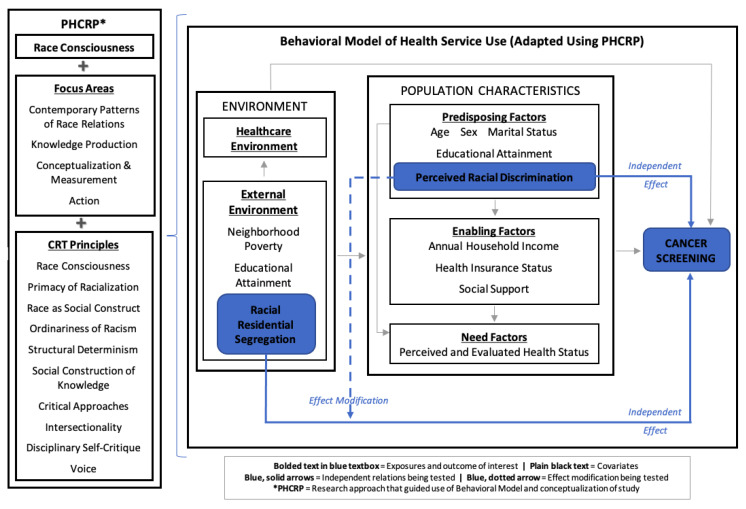
Conceptual framework: adapted behavioral model of health service use using public health critical race praxis.

The Public Health Critical Race Praxis (PHCRP) research approach [48] and the Behavioral Model of Health Services Use [49,50,51] informed the conceptual framework for this study (Figure 1). PHCRP is an iterative methodology, grounded in critical race theory (CRT), that emphasizes a race-consciousness approach to research, that is, being aware of and explicating the ways in which racism may be operating [52]. The behavioral model posits causal associations between key individual-level and contextual determinants, such as predisposing factors that incline or disincline individuals to use care (i.e., age, sex, marital status, and individual educational attainment), enabling factors that enable or impede use of care (i.e., income and insurance status), need factors that indicate the extent of individuals’ perceived or evaluated need for care (i.e., perceived and evaluated health status), and environmental factors (e.g., neighborhood poverty and educational attainment).

The behavioral model guided hypothesized associations between key individual-level and contextual determinants in this study, while PHCRP’s race-consciousness approach guided conceptualization and operationalization throughout the research process. For example, although Black/African American race is frequently examined within the behavioral model (and disparities research as a whole) as a risk factor for poor health (i.e., a proxy for racism), informed by PHCRP, we excluded race as an exposure and restricted our study sample to African Americans. This shifts the focus of the study from a question of “how race influences cancer screening” to one that explicitly examines the influence of racialized experiences among African Americans (i.e., experiences of racial discrimination and living in a racially segregated neighborhood) on their health outcomes [48,53]. The analyses in this study examined a subset of the relations depicted in Figure 1, specifically, the independent and interactive effects of perceived racial discrimination (predisposing factor) and racial residential segregation (external environmental exposure) on cancer screening. Other factors informed by the behavioral model were included as covariates.

### 2.3. Study Setting and Participants

The 2-1-1 Texas/United Way Helpline (also referred to as the Gulf Coast Regional 2-1-1 Texas Area Information Center [AIC or call center]) based in Houston, TX is the largest 2-1-1 helpline in the country. They receive an average of 60,600 calls per month from residents across the region. Procedures for participant recruitment in the parent study are detailed elsewhere [54]. Briefly, between February 2011 and May 2013, we randomly selected callers aged 18 years or older from all callers to the Gulf Coast Regional call center and invited them to complete a cancer risk assessment to assess their eligibility for participating in the parent study. Ultimately, we enrolled 1554 eligible callers. Of those, 52% (*n* = 866) were administered the perceived discrimination scale (employed a planned missingness approach [55] to reduce survey burden), and 56% (*n* = 483) of this group were African American and, thus, eligible for inclusion in the current study. Of these 483 African Americans who received the perceived discrimination scale, 334 were screened out for not meeting the following inclusion criteria: (1) completion of a follow-up survey, (2) being female, and (3) being non-adherent at baseline to either Pap test, mammography, or colorectal cancer screening recommendations in place at the time of the study according to the American Cancer Society [56] (e.g., either had not had a Pap test within the last year, had not had a mammogram within the last year, and/or had not completed a home-based stool test within the last year, sigmoidoscopy within the last five years, or colonoscopy within the last ten years). Given these criteria, there were 149 female participants available for analysis of the current study questions (that is, 31% of all African Americans who were administered the perceived racial discrimination scale within the parent study). 

### 2.4. Data Collection

We used individual-level, self-report baseline and follow-up data collected between February 2011 and June 2015 as part of the parent study. We also obtained Greater Houston TX metropolitan statistical area (MSA) and census tract population values from the US Census Bureau’s 2011–2015 American Community Survey 5-Year Estimates to compute levels of racial residential segregation, poverty, and educational attainment [57,58,59]. These data were appended to the individual-level data. 

#### 2.4.1. Cancer Screening Outcome

The primary outcome in this study was “any cancer screening completion” (or uptake), a binary variable (0 = did not complete, 1 = completed) indicating whether participants completed at follow-up at least one needed cancer screening based on the baseline determination of non-adherence. 

#### 2.4.2. Indicators of Racism

We used two indicators of racism assessed at baseline: perceived racial discrimination (independent variable for Hypothesis 1 and effect modifier for exploratory analysis) and racial residential segregation (independent variable for Hypothesis 2 and focal predictor for exploratory analysis).

##### Perceived Racial Discrimination

We assessed perceived racial discrimination using a two-step method. First, we administrated to participants a modified version of the validated 9-item Experiences of Discrimination (EOD) scale (Cronbach’s *α* = 0.81) [38]. Using a yes/no scale, 2-1-1 information specialists asked participants to indicate whether they had experienced discrimination, been prevented from doing something, or been hassled or made to feel inferior in nine different situations in the last five years. The situations included: (1) while at school; (2) when being hired or obtaining a job; (3) while at work; (4) when obtaining housing; (5) in accessing or while obtaining medical care; (6) when obtaining service in a store or restaurant; (7) when obtaining credit, bank loans, or a mortgage; (8) while on the street or in a public setting; (9) from the police or in the courts. 

Next, if participants answered “yes” to any of the nine situations indicated above, using a single item, they were asked to indicate what they thought was the main reason for their collective experiences. The reasons included: (1) ancestry or national origin, (2) gender, (3) race/ethnicity, (4) shade of skin color, (5) age, (6) religion, (7) sexual orientation, (8) education or income level, (9) physical disability, or (10) other. We reviewed the “other” category and recoded all responses that indicated ancestry or national origin (coded 1), race/ethnicity (coded 3), and/or shade of skin color (coded 4). If an individual indicated more than one reason or a reason not among those listed, the reason was coded as “other.”

For each participant who reported racial discrimination (i.e., race/ethnicity, ancestry or national origin, and/or shade of skin color indicated as the main reason for their collective experiences), we calculated a sum of all situations in which they indicated they had experienced discrimination (i.e., number of items affirmed within the 9-item scale). A summary score of zero was assigned to those who reported no experiences of discrimination or who reported the main reason for the discrimination as something other than race/ethnicity, ancestry or national origin, or shade of skin color. Due to the non-normal distribution of the variable, with more than 50% of the sample as having a summary score of zero and the remaining as having a score of 1 or more, we generated a binary variable. For those who reported some other discrimination or none, we categorized the level of perceived racial discrimination as “no perceived racial discrimination”. Reports of experiencing racial discrimination in at least one of the nine situation types were categorized as “perceived racial discrimination”.

##### Racial Residential Segregation

Contemporary residential racial segregation within urban areas, while in part influenced by neighborhood preferences, largely reflects the impact of many interrelated processes historically rooted in racism (e.g., Jim Crow segregation, red-lining, and mortgage lending bias) [60,61,62] and is often unhealthy for African Americans [23,63]. We measured racial residential segregation within participants’ neighborhoods, defined at the census-tract level, using the Location Quotient for Racial Residential Segregation (LQRRS) [63]. The LQRRS is a local area measure of relative segregation that quantifies the relative racial homogeneity of a residential neighborhood (i.e., census tract) compared to the racial homogeneity within the larger MSAs in which the census tract is located. It is a ratio of two proportions that indicates how much more segregated an individual’s neighborhood is relative to the MSA; that is, the proportion of African Americans who reside in a neighborhood (numerator) and the proportion of African Americans who reside in the MSA (denominator) [63,64]. The LQRRS can be calculated for any two groups or characteristics (e.g., Black–White segregation, Black–other segregation), and it is the unevenness or relative differences that are important to measure when assessing racial residential segregation [23,64]. The LQRRS is a relative measure of residential segregation that considers a local community within the confines of a larger metropolitan statistical area or urbanized region. 

For this study, we assessed racial residential segregation for African Americans vs. all other racial groups (i.e., Black–Non-Black residential segregation). We identified the census tract and MSA location for each participant, using the US Census Bureau Geocoder based on 2010 census tract and MSA delineations (the Census year closest to when residential data were collected from participants) and obtained census tract and MSA population values from the US Census Bureau [57].

We created a continuous variable using the following the LQRRS equation: LQRRS_i_ = (b_i_/t_i_)/(B/T), where LQRRS_i_ is the level of Black–Non-Black segregation within a neighborhood relative to the larger MSA; b_i_ is the total number of African Americans who live within a neighborhood; t_i_ is the total number of residents who live within a neighborhood (all racial groups); B is the total number of African Americans who live in the MSA; T is the total number of residents who live in the MSA. Following similar studies of segregation, we categorized the LQRRS to facilitate interpretation [63,65]. An LQRRS of 1.2 or greater was categorized as high Black segregation (i.e., overrepresentation of African Americans in a neighborhood in comparison to their representation in the larger MSA), an LQRSS less than 1.2 and greater than 0.85 is categorized as integrated (i.e., equal representation), and an LQRSS of 0.85 or less is categorized as high Non-Black segregation (i.e., under-representation of African Americans in a neighborhood in comparison to their representation in the larger MSA). These thresholds roughly correspond with one standard deviation above or below LQRSS = 1.0 [66]. Based on the non-normal distribution of the variable, the LQRRS variable was dichotomized by collapsing the integrated and high Non-Black segregation categories into a single “no high Black segregation” category (hereafter referred to as “not living in a segregated neighborhood”) versus the “high Black segregation” category (hereafter referred to as “living in a segregated neighborhood”). Ultimately, this categorization aligned with the question we aimed to investigate, that is, how Black segregation vs. no Black segregation relates to cancer screening behavior.

#### 2.4.3. Covariates 

In alignment with our conceptual framework, we accounted for predisposing, enabling, need, and environmental covariates in our analyses. Predisposing factors considered were age (in years), educational attainment (less than high school; high school or GED; post-high school [i.e., vocational, technical, or associates degree, some college, bachelor’s degree, or higher]), and marital status (not married or living with someone; married or living with someone). Enabling factors considered were annual household income (less than USD 10,000; USD 10,000–USD 19,999; USD 20,000 or more; categories align with distribution of the data) and insurance status (no insurance or the Children’s Health Insurance Plan only; public and/or private insurance). We accounted for need factors during the selection of eligible participants (i.e., all participants had to be in need of at least one screening test to be eligible for the study). In addition, we generated a variable to adjust for the number of screening tests that a participant needed. Environmental factors considered were neighborhood poverty (% of residents who live in poverty in the census tract) and educational attainment (% of residents aged 25 and older who had graduated from high school and % of residents aged 25 and older who had earned a bachelor’s degree) obtained from the US Census Bureau [58,59]. Given that this study was a secondary data analysis of an intervention study, we included intervention status (participants received a referral to cancer screening only; participants received a referral to cancer screening plus patient navigation) as a covariate in all adjusted models to take into account intervention effects. 

### 2.5. Data Analysis

Prior to conducting the main analyses, we examined the levels of missing data on the independent variables and covariates and found that they were not problematic (criterion: <5% missing per variable) [67]; thus, we employed a complete case analysis approach. To assess potential multicollinearity, we examined correlations between the indicators of racism and other covariates (i.e., predisposing factors, enabling, need, and environmental factors). We also examined bivariate relations between each covariate and any cancer screening completion. Covariates that were associated with the outcome in bivariate analyses and that did not exhibit high collinearity with the indicators of racism or other covariates (criterion: variance inflation factor < 10) [68] were entered into the multivariable analyses below. Intervention status was included as a covariate in all adjusted models. 

We fit multivariable logistic regression models using generalized estimating equations (GEE) to adjust for clustering at the census-tract level. GEE is the preferred multilevel approach when the neighborhood-level units are not a random sample of a larger universe of census tracts [53,69]. We specified an exchangeable correlation structure and robust standard errors.

To test Hypothesis 1, we entered into the model perceived racial discrimination as the independent variable and any cancer screening as the outcome variable. Then, we adjusted the model by simultaneously adding the intervention status variable and covariates associated with any cancer screening completion in bivariate analyses at *p* < 0.25 level [70]. To test Hypothesis 2, the same procedures were performed, using racial residential segregation as the independent variable. To explore the potential modifying effect of discrimination on the relation between segregation and screening, we entered perceived racial discrimination, racial residential segregation, any cancer screening completion, and an interaction term between discrimination and segregation into an initial model. Then, this model was adjusted by simultaneously adding the intervention status variable and covariates associated with any cancer screening completion. For all models, we conducted appropriate data diagnostics (e.g., checking for linearity in the logit) to ensure that there were no violations to the logistic regression model assumptions. Stata/SE version 16 [71] was used to conduct all analyses. We set the alpha threshold as *α* < 0.25 (two-tailed) [70] for bivariate analyses and *α* < 0.05 (two-tailed) for main effects (multivariable analyses). Given our relatively small sample size and the exploratory nature of our test for effect modification, we set *α* at <0.20 (two-tailed) for interaction and stratified analyses [72] as a threshold for rejection only (not to make statements about statistical significance) so as not to miss potentially meaningful interactions that could generate hypotheses for future studies.

## 3. Results

### 3.1. Descriptive Statistics

Table 1 presents the individual- and neighborhood-level characteristics of the sample at baseline. The sample consisted of 149 African American women in need of a Pap test, mammogram, and/or colorectal cancer screening. Their average age was 48 years, and most were not married (89.2%), had a post-high school education (48.3%), reported an annual household income of less than USD 10,000 (40.9%), and had public and/or private insurance (57.1%). At baseline, 45.0% reported that they had experienced racial discrimination. 

As shown in Table 2, the situations reported most frequently as places where participants experienced racial discrimination were “when getting service in a store or restaurant” (71.6%) and “while at work” (56.7%). More than a third (34.3%) reported discrimination “in accessing or while getting medical care.” On average, participants needed 1.6 cancer screening tests at baseline. A little more than half of the sample (56.4%) reported completing at least one needed cancer screening at follow-up (Table 3).

Women resided within 119 unique neighborhoods (i.e., census tracts) in the Houston–The Woodlands–Sugar Land, TX, MSA (hereafter referred to as Greater Houston MSA). On average, nearly 30% of the population aged 25 and older in these neighborhoods were high school graduates (includes equivalency) and about 12% had a bachelor’s degree (Table 1). About 25% of the population within these neighborhoods had household incomes below the federal poverty level. A majority of study participants (67.8%) lived in a segregated neighborhood.

### 3.2. Perceived Racial Discrimination and Cancer Screening

We tested the hypothesis that there is a significant negative relation between reporting experiences of racial discrimination at baseline and obtaining any cancer screening at follow-up among the sample of African American women who were non-adherent to at least one screening at baseline. Table 4 presents the unadjusted OR and adjusted OR (aOR), *p*-values, and 95% confidence intervals (CI) for this relation. Contrary to the hypothesis, a statistically significant positive relation was found between perceived racial discrimination and any cancer screening completion (aOR = 2.79, 95% CI: 1.37–5.67). That is, women who reported racial discrimination had, on average, 2.79 times greater odds of completing any cancer screening at follow-up compared to women who did not report racial discrimination. None of the covariates included in the adjusted model (age, education, annual household income, insurance status, total cancer screenings needed, or intervention status) showed a statistically significant association with any cancer screening completion. 

### 3.3. Racial Residential Segregation and Cancer Screening

We tested the hypothesis that there was a significant negative relation between living in a segregated neighborhood and obtaining any cancer screening at follow-up. Table 4 provides the unadjusted OR and aOR, *p*-values, and 95% CIs for this relation. Although the direction of the relation between racial residential segregation and any cancer screening completion at follow-up was in the negative direction (aOR = 0.89, 95% CI: 0.44–1.79), the result was not significant, and the confidence interval suggests the potential for different directionalities of the relation. None of the covariates included in the adjusted model were statistically significant.

### 3.4. Modifying Effect of Perceived Racial Discrimination on Racial Residential Segregation and Cancer Screening

**Figure 2:**
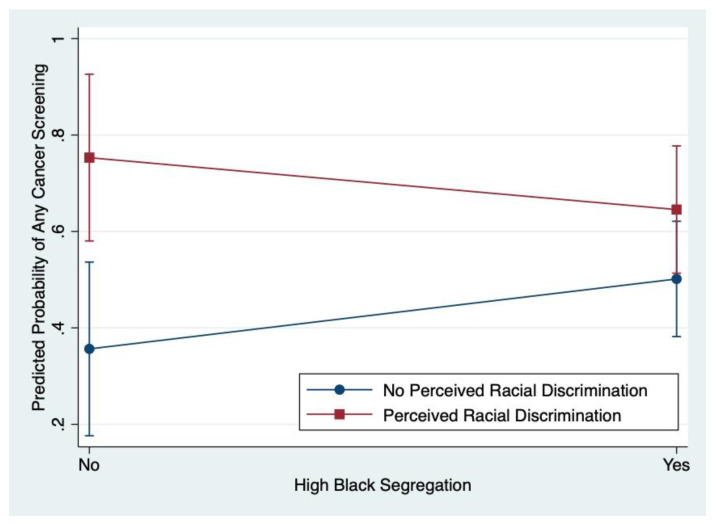
Modifying effect of perceived racial discrimination on racial residential segregation and any cancer screening completion.

We conducted exploratory analyses to determine whether perceived racial discrimination modified the relation between racial residential segregation and any cancer screening at follow-up among African American women in the sample. Using our exploratory alpha threshold, *α* < 0.20, there was some evidence of a qualitative interaction between discrimination and segregation (*p* = 0.117); that is, as shown in Figure 2, the direction of the relation between segregation and cancer screening appears to change from positive to negative when taking into consideration perceived racial discrimination as an effect modifier. Thus, the results for the relation between racial residential segregation and any cancer screening were stratified by perceived racial discrimination for further examination (Table 5). 

These stratified analyses did not meet the alpha threshold (*α* < 0.20) set for these exploratory analyses, likely due to the small sample sizes in each stratum, and the confidence intervals suggest the potential for different directionalities. However, the results may suggest that among those who do not report perceived racial discrimination at baseline, African women who live in a segregated neighborhood may have greater odds of obtaining any cancer screening at follow-up compared to women who did not live in a segregated neighborhood (aOR = 1.90, 95% CI: 0.66–5.45, *p* = 0.231). In addition, among women who do report perceived racial discrimination at baseline, those who live in a segregated neighborhood may have lower odds of completing any cancer screening at follow-up compared to women who do not live in a segregated neighborhood (aOR = 0.474, 95% CI: 0.13–1.72, *p* = 0.256). 

## 4. Discussion

Although racism is increasingly being examined as an important contributor to racial and ethnic health inequities, its relation to cancer-related behaviors and outcomes among African Americans remains unclear. The purpose of this study was to clarify the relation between two indicators of racism—perceived racial discrimination and racial residential segregation—and cancer screening. Although not in the hypothesized direction, we did find a significant longitudinal relation between perceived racial discrimination at baseline and cancer screening completion at follow-up among African American women in the sample. This finding differs from much of the current literature, which has been primarily cross-sectional and has often reported negative associations between perceived racial discrimination and cancer screening [26,27,29,30,32,33] or no association at all [27,28,31,32,33,34,35]. 

The positive relation we found, however, is similar to that of a cross-sectional study conducted by Benjamins et al. [31] on the association between different types of cancer screening (cervical, breast, colorectal, and prostate) and perceived racial discrimination, using a healthcare discrimination measure, the Everyday Discrimination Scale, and the Experiences of Discrimination scale used in this study. This study found a significant positive association between clinical breast examination and everyday discrimination.

In our study, the relation between racial residential segregation and any cancer screening completion at follow-up was in the negative direction; however, the results were not statistically significant. Although literature on this topic is limited, two of the three studies found examining the association between racial residential segregation and cancer screening describe a negative association between segregation and mammography use [43] and colorectal cancer screening [44]), while the third study suggests a positive relation between segregation and mammography use [45]. The lack of clear directionality between segregation and cancer screening completion in our study could be the result of our use of a combined any cancer screening outcome, which included mammography, colorectal cancer screening, and Pap test completion. 

At conceptualization of this study, we planned to explore potential mechanisms underlying the relations examined in our primary aims; specifically, we examined whether there was an interaction between perceived racial discrimination and racial residential segregation. This investigation revealed some preliminary evidence of a qualitative interaction, meaning that the direction of the association between segregation and cancer screening may vary depending on the presence or absence of discrimination. Visual inspection of a graphical representation of this relation as well as examination of stratified regression results suggest that living in a segregated neighborhood may be negatively related to cancer screening behavior for women who report experiences of racial discrimination but positively related for women who do not report racial discrimination. These exploratory analyses reveal important patterns of results that merit attention even though they do not reach the conventional significance level. Notably, this finding may indicate that racial discrimination is a potential driver for how residential segregation leads to non-participation in healthful behaviors such as cancer screening.

Aspects of the above findings align with similar research conducted by Borrell et al. [46], who investigated the association between perceived racial discrimination and multiple health behaviors (i.e., smoking, alcohol consumption, and physical activity) as well as whether these relations were modified by racial residential segregation. Similar to our study, discrimination and segregation were negatively related, and discrimination was positively related to one of the health behaviors they examined (physical activity). The underlying mechanisms of the relation between discrimination and physical activity in their study, however, likely differ from those examined in our study (e.g., physical activity may be a coping mechanism for experiences of discrimination, thus explaining their positive association). In contrast to our study, Borrell et al. [46] reported that there was no significant interaction between discrimination and segregation for any of the health behaviors examined. The interactive relation found in our study warrants further examination in future well-powered studies. 

Our study found that African American women who report more racial discrimination are more likely to be screened, and African American women living in segregated neighborhoods are less likely to be screened. One explanation of these findings is that African Americans who live in less segregated neighborhoods (i.e., more integrated neighborhoods) [46,47], with more racial diversity, have more exposure (opportunity) to be discriminated against. At the same time, these women who live in more integrated neighborhoods may be more comfortable going into healthcare facilities that are more racially diverse, like their living circumstances, even though they might experience some racial discrimination in the process. Conversely, African American women who live in more segregated neighborhoods (but experience less discrimination), may be less likely to access services outside of their communities for various reasons including higher levels of medical mistrust [73]. Other reasons for differences in screening in more segregated versus less segregated neighborhoods may include differential access to screening such as more or less availability and accessibility of healthcare facilities as well as quality factors (i.e., health system infrastructure, provider characteristics) [73,74]. Finally, African American women who live in more integrated neighborhoods and report greater levels of racial discrimination may also have a greater sense of race consciousness, which may be positively related to health consciousness and an orientation towards self-protective behavior [75,76] that translates to greater levels of cancer screening. 

Our study is the first to investigate the potential modifying effect of discrimination and segregation on cancer screening behavior among African Americans, and our novel findings may help to explain the mixed results often seen when examining independent associations between discrimination and segregation with cancer screening. Notably, the interaction found in our study suggests that, to better understand how racism affects cancer-screening behavior, it is important to consider the larger context in which African Americans live, work, and play. Despite increasing interest in the role of residential neighborhoods, few cancer screening studies have examined how contextual factors, such as racial residential segregation, influence cancer screening behavior or how other racialized experiences, such as discrimination, affect this relation. We observed that a substantial proportion of our sample experienced racial discrimination (42%), although it was lower than the rate reported in a recent population-based study of perceived racial discrimination among African Americans (70% vs. 42% in our study). In addition, a majority of women in our sample lived in segregated neighborhoods. These findings suggest that these two indicators of racism are prevalent among low-income, medically underserved African American women but still have largely unknown impacts across the cancer continuum. This points to the need for more study of the associations examined here as well as how they can be addressed through intervention. In addition, given this sample represents a group that is particularly vulnerable to cancer-related disparities, i.e., medically underserved, low-income, and a racial minority group, it may be important to examine how intersectionality (combined effects of racism, sexism, and classism) impacts the relations examined in this study. Future studies with sufficient sample sizes should be conducted to confirm and expand on our findings, preferably for Pap test screening, mammography, and colorectal cancer screening separately. 

There are a few limitations of this study. First, this was a secondary data analysis of a larger intervention study that was not population based. Thus, the findings in our study cannot be generalized to African Americans broadly, as the sample consisted of low-income, medically underserved women who lived in Texas. In addition, those who call 2-1-1 may be different from those who do not. Because participants had to be non-adherent to at least one cancer prevention service to be included in the parent study, the current study may have some form of selection bias from attenuation of the range of exposure. We also only included those who completed a follow-up survey so they may be different in unknown ways from those who did not complete the follow-up survey. In addition, self-reported outcome data may be vulnerable to recall bias and social desirability. We dichotomized the perceived racial discrimination variable given that the distribution was highly skewed, with over 50% of respondents with a value of zero (0). We also dichotomized the segregation variable due to its non-normal distribution and the inability to normalize it via transformation. Collapsing categories within these variables may have contributed to a loss in potentially valuable information; however, we reasoned that the qualitative difference between those that faced no discrimination vs. those that reported any discrimination (and those who lived in a Black segregated neighborhood vs. not) was more important than the quantitative difference across successive values of each scale. Finally, given the fixed sample size, power was limited in detecting associations of interest. 

Despite its limitations, this study has many strengths that make it a significant contribution to the literature. One major strength is that a racial equity approach, specifically PHCRP [48], informed the research process. One way in which this approach was applied was by restricting the sample to African Americans. In cancer prevention and control research and public health research more broadly, health among racial and ethnic minorities is often compared to Whites as the standard. Such comparisons between races lead us to answer questions such as, “How does Black race influence cancer screening?” [53,77], rather than racial equity-focused questions, such as, “How do the racialized experiences of African Americans influence cancer screening?” [53,77]. Health equity and disparities researchers often try to understand and address the latter, and restricting the sample to African Americans facilitates this inquiry. Few studies have tested the association between racial residential segregation and cancer screening specifically among African Americans, and, to the authors’ knowledge, none had considered Pap test screening, and only one had examined colorectal cancer screening. In addition, researchers have called for increased examination of potential mechanisms that underlie the relation between racism and health, in particular, discrimination and health [22,41]. Ours is the first study, to our knowledge, to use multiple measures of racism to investigate the association between racism and cancer screening, including the modifying effect of perceived racial discrimination on the racial residential segregation-cancer screening link. Furthermore, it focuses on a group that is especially vulnerable to cancer-related disparities due to their multiple minority and disadvantaged status, i.e., low-income, medically underserved, and a racial minority group. This is also the first study to examine a combined cancer screening uptake outcome and its association with perceived racial discrimination and racial residential segregation. Another strength is that we used longitudinal data and multilevel modeling. There have been calls for this level of methodological rigor in the examination of racism and health [22,27,33,41]; yet, there is only one known study that has used longitudinal data and multilevel modeling to examine the relation between perceived racial discrimination and cancer screening among African Americans, and no studies have used longitudinal data to examine racial residential segregation and cancer screening among African Americans. Finally, we used a validated, multi-item measure to assess perceived racial discrimination, which tends to be lacking in the literature [36,37,38,39,40], and a local area, relative measure of racial residential segregation, in contrast to many studies that use measures such as the dissimilarity index that can measure segregation at only larger (MSA or county level) levels [42,63,64,78]. 

## 5. Conclusions

This study is among the first to examine, longitudinally, how racism, as measured by perceived racial discrimination and racial residential segregation, is independently and interactively related to cancer screening completion among African Americans. Our findings suggest that racism is associated with cancer screening but that these relations are nuanced and still somewhat uncertain. Perceived racial discrimination and racial residential segregation should be considered jointly, rather than independently, to better understand their influence on screening behavior. Future studies with sufficient samples sizes should be conducted to confirm our findings, investigate how the combined effects of racism, sexism, and classism may impact the relations examined in this study, and test anti-racist interventions.

## Figures and Tables

**Table 1:** Characteristics of African Americans in the sample and bivariate association with indicators of racism (*n* = 149).

INDIVIDUAL-LEVEL CHARACTERISTICS					
	Total Sample	Perceived Racial Discrimination ^1^		Racial Residential Segregation ^1^	
		No (*n* = 82)	Yes (*n* = 67)	No (*n* = 48)	Yes (*n* = 101)
**Age (years), mean (SD)**	48.1 (11.4)	49 (10.1)	47.0 (12.8)	49.1 (9.1)	47.7 (12.3)
		*p* = 0.2978		*p* = 0.4779	
**Marital Status, *n* (%)**					
Not Married	132 (89.2)	73 (89.0)	59 (89.4)	43 (89.6)	89 (89.0)
Married or Living with a Someone	16 (10.8)	9 (11.0)	7 (10.6)	5 (10.4)	11 (11.0)
		*p* = 0.943		*p* = 0.915	
**Education, *n* (%)**					
Less than High School	21 (14.1)	12 (14.6)	9 (13.4)	8 (16.7)	13 (12.9)
High School or GED	56 (37.6)	32 (39.0)	24 (35.8)	17 (35.4)	39 (38.6)
Post High School ^2^	72 (48.3)	38 (46.3)	34 (50.8)	23 (47.9)	49 (48.5)
		*p* = 0.867		*p* = 0.809	
**Annual Household Income, *n* (%)**					
None–USD 9999	61 (40.9)	37 (45.1)	24 (35.8)	23 (47.9)	38 (37.6)
USD 10,000–USD 19,999	53 (35.6)	25 (30.5)	28 (41.8)	16 (33.3)	37 (36.6)
USD 20,000 or more	35 (23.5)	20 (24.4)	15 (22.4)	9 (18.8)	26 (25.7)
		*p* = 0.338		*p* = 0.445	
**Health Insurance Status, *n* (%)**					
No Insurance (or CHIP only)	64 (43.0)	35 (42.7)	29 (43.3)	20 (41.7)	44 (43.6)
Public and/or Private Insurance	85 (57.1)	47 (57.3)	38 (56.7)	28 (58.3)	57 (56.4)
		*p* = 0.941		*p* = 0.827	
**Number of Cancer Screenings Needed, mean (SD)**	1.6 (0.7)	1.7 (0.73)	1.6 (0.72)	1.9 (0.8)	1.5 (0.7)
		*p* = 0.3368		*p* = 0.0045 **	
**Social Support**					
Functional Social Support Scale Score, mean (SD)	22.8 (5.8)	23.1 (5.9)	22.5 (5.7)	23.3 (5.4)	22.6 (6.0)
		*p* = 0.5221		*p* = 0.5003	
Levels of Perceived Functional Social Support, *n* (%)					
Low	30 (28.3)	22 (26.8)	18 (26.9)	10 (28.6)	20 (28.2)
Moderate/High	76 (71.7)	60 (73.2)	49 (73.1)	25 (71.4)	51 (71.8)
		*p* = 0.996		*p* = 0.965	
**Perceived Racial Discrimination**					
Reported Experiences of Racial Discrimination, *n* (%)					
No	82 (55.0)	-	-	21 (43.8)	61 (60.4)
Yes	67 (45.0)	-	-	27 (56.3)	40 (39.6)
		-		*p* = 0.056 ^†^	
Number of Situations Reported in the Last 5 Years, mean (SD)	1.6 (2.2)	-	-	2.0 (2.3)	1.5 (2.1)
		-		*p* = 0.1875 ^†^	
**NEIGHBORHOOD-LEVEL CHARACTERISTICS**					
**Educational Attainment**					
Percent Neighborhood with High School/GED ^3^, mean (SD)	29.0 (8.7)	28.4 (8.7)	29.8 (8.7)	25.8 (8.9)	30.6 (8.2)
		*p* = 0.3310		*p* = 0.0016 **	
Percent Neighborhood with Bachelor’s Degree ^4^, mean (SD)	12.1 (9.2)	11.7 (8.1)	12.6 (10.3)	13.3 (12.4)	11.5 (7.1)
		*p* = 0.5255		*p* = 0.2522	
**Percent Neighborhood Below Federal Poverty Line, mean (SD)**	25.2 (11.9)	25.4 (11.6)	24.8 (12.3)	25.0 (14.2)	25.2 (10.7)
		*p* = 0.7457		*p* = 0.9229	
**Racial Residential Segregation**					
Location Quotient (unitless), mean (SD)	2.3 (1.5)	2.6 (1.6)	2.0 (1.4)	-	-
		*p* = 0.0195 *		-	
Participants Living in High Black Segregated Neighborhood, *n* (%)					
No	48 (32.2)	21 (25.6)	27 (40.3)	-	-
Yes	101 (67.8)	61 (74.4)	40 (59.7)	-	-
		*p* = 0.056 ^†^		-	

CHIP: Texas Children’s Health Plan available to low-income pregnant women who do not qualify for Medicaid and do not have health insurance. ^1^ Pearson Chi-squared test (two-tailed) for categorical variables and independent samples *t*-test (two-tailed) for continuous variables. ^2^ Includes vocational, technical, or associate degree, some college, and bachelor’s degree or higher. ^3^ Percentage of the population 25 years and older that is high school/GED graduate. ^4^ Percentage of the population 25 years and older that has bachelor’s degree. † *p* < 0.25, two-tailed. * *p* < 0.05, two-tailed. ** *p* < 0.01, two-tailed.

**Table 2:** Experiences of racial discrimination reported (*n* = 67).

Situtation Types	Total Sample *n* (%)
When gettting service in a store or restaurant	48 (71.6)
While at work	38 (56.7)
When getting hired or getting a job	31 (46.3)
While on the street or in a public setting	30 (44.8)
From the police or in the courts	27 (40.3)
When getting credit, bank loans, or a mortgage (*n* = 48)	17 (35.4)
In accessing or while getting medical care	23 (34.3)
When getting housing	19 (29.2)
While at school	10 (14.9)

**Table 3:** Any cancer screening completion at follow-up (*n* = 149).

	Total Sample
Did Not Complete Any Needed Screening, *n* (%)	65 (43.6)
Completed at Least One Needed Screening, *n* (%)	84 (56.4)
Number of Cancer Screenings Completed, mean (SD)	0.80 (0.87)

Any cancer screening completion: Participant completed at least one of the following cancer screenings for which they were eligible: Pap test, mammography, colorectal cancer screening.

**Table 4:** Unadjusted and adjusted odd ratios for the association between each indicator of racism at baseline and any cancer screening completion at follow-up (*n* = 149).

	Unadjusted Bivariate Models	Adjusted Multivariable Models ^1^	
		Discrimination Model	Segregation Model
INDICATORS OF RACISM	OR (95% CI)	aOR (95% CI)	aOR (95% CI)
**Perceived Racial Discrimination**			
No	Reference	Reference	Not Included
Yes	2.56 (1.31–4.97) **	2.79 (1.37–5.67) **	Not Included
**Racial Residential Segregation** **(High Black Segregation)**			
No	Reference	Not Included	Reference
Yes	0.88 (0.44–1.76)	Not Included	0.89 (0.44–1.79)
**COVARIATES**			
**Age (years)**	1.02 (0.99–1.05) ^†^	1.00 (0.96–1.04)	1.00 (0.96–1.04)
**Marital Status**			
Not Married	Reference	Not Included	Not Included
Married or Living with a Someone	0.57 (0.20–1.63)	Not Included	Not Included
**Education**			
Less than High School	Reference	Reference	Reference
High School or GED	2.02 (0.72–5.62) ^†^	1.84 (0.58–5.83)	1.84 (0.62–5.44)
Post High School ^2^	2.71 (0.99–7.37) ^†^	2.43 (0.74–7.95)	2.49 (0.82–7.57)
**Annual Household Income**			
None–USD 9999	Reference	Reference	Reference
USD 10,000–USD 19,999	1.46 (0.69–3.06)	1.50 (0.70–3.24)	1.74 (0.84–3.61)
USD 20,000 or more	1.98 (0.84–4.68) ^†^	2.07 (0.83–5.16)	2.14 (0.85–5.36)
**Health Insurance Status**			
No Insurance (or CHIP only)	Reference	Reference	Reference
Public and/or Private Insurance	1.58 (0.82–3.04) ^†^	1.88 (0.84–4.19)	1.88 (0.85–4.15)
**Number of Cancer Screenings Needed**	1.53 (0.96–2.43) ^†^	1.60 (0.92–2.79)	1.49 (0.87–2.56)
**Neighborhood Educational Attainment**			
Percent with High School/GED ^3^	1.00 (0.96–1.03)	Not Included	Not Included
Percent with Bachelor’s Degree ^4^	0.99 (0.95–1.02)	Not Included	Not Included
**Percent Neighborhood Below Federal Poverty Line**	0.99 (0.97–1.02)	Not Included	Not Included

OR, odds ratio; aOR, adjusted odds ratio; 95% CI, 95% confidence interval CHIP: Texas Children’s Health Plan available to low-income pregnant women who do not qualify for Medicaid and do not have health insurance. ^1^ Model adjusted for intervention status to control for intervention effects due to nature of the parent study from which these data were obtained for secondary analysis. Intervention did not have statistically significant effect in either model (results not shown). ^2^ Includes vocational, technical, or associate degree, some college, and bachelor’s degree or higher. ^3^ Percentage of the population 25 years and older that is high school/GED graduate. ^4^ Percentage of the population 25 years and older that has bachelor’s degree. † *p* < 0.25, two-tailed (unadjusted results only). ** *p* < 0.01, two-tailed.

**Table 5:** Effect modification: adjusted odd ratios for relation between racial residential segregation and any cancer screening completion at follow-up stratified by perceived racial discrimination at baseline (*n* = 149).

	Perceived Racial Discrimination Strata ^1^	
	No (*n* = 82)	Yes (*n* = 67)
EXPOSURES OF INTEREST	aOR (95% CI)	aOR (95% CI)
**Racial Residential Segregation** **(High Black Segregation)**		
No	Reference	Reference
Yes	1.90 (0.66–5.45)	0.47 (0.13–1.72)
**Age (years), mean (SD)**	0.97 (0.91–1.03)	1.02 (0.96–1.08)
**Education**		
Less than High School	Reference	Reference
High School or GED	0.56 (0.12–2.54)	7.30 (1.33–40.00) *
Post High School ^2^	1.13 (0.26–4.95)	5.87 (0.91–37.72) ^††^
**Annual Household Income**		
None–USD 9999	Reference	Reference
USD 10,000–USD 19,999	0.89 (0.29–2.74)	1.72 (0.42–7.03)
USD 20,000 or more	2.49 (0.75–8.27) ^††^	2.15 (0.43–10.75)
**Health Insurance Status**		
No Insurance (or CHIP only)	Reference	Reference
Public and/or Private Insurance	3.51 (1.12–10.96) *	0.92 (0.29–2.98)
**Number of Cancer Screenings Needed**	2.02 (0.88–4.64) ^††^	2.00 (0.70–5.71) ^††^

OR, odds ratio; aOR, adjusted odds ratio; 95% CI, 95% confidence interval CHIP: Texas Children’s Health Plan available to low-income pregnant women who do not qualify for Medicaid and do not have health insurance. ^1^ Models adjusted for intervention status to control for intervention effects due to nature of the parent study from which these data were obtained for secondary analysis. Intervention did not have statistically significant effect in either model (results not shown). ^2^ Includes vocational, technical, or associate degree, some college, and bachelor’s degree or higher. †† *p* < 0.20, two-tailed (interaction/stratified analyses only). * *p* < 0.05, two-tailed.

## Data Availability

Data are available upon request from the corresponding author.
